# Screening of immune-related secretory proteins linking chronic kidney disease with calcific aortic valve disease based on comprehensive bioinformatics analysis and machine learning

**DOI:** 10.1186/s12967-023-04171-x

**Published:** 2023-06-01

**Authors:** Enyi Zhu, Xiaorong Shu, Zi Xu, Yanren Peng, Yunxiu Xiang, Yu Liu, Hui Guan, Ming Zhong, Jinhong Li, Li-Zhen Zhang, Ruqiong Nie, Zhihua Zheng

**Affiliations:** 1 Department of Nephrology, Center of Kidney and Urology, The Seventh Affiliated Hospital, Sun Yat-sen University, Shenzhen, China; 2 Department of Cardiology, Sun Yat-sen Memorial Hospital of Sun Yat-sen University, Guangzhou, China; 3 Department of Radiology, Guizhou Provincial People’s Hospital, Guizhou, China; 4 Department of Urology, The First Affiliated Hospital of Sun Yat-sen University, Guangzhou, China

**Keywords:** Chronic kidney disease, Calcific aortic valve disease, Immune cell infiltration, Diagnostic value, Secretory proteins

## Abstract

**Background:**

Chronic kidney disease (CKD) is one of the most significant cardiovascular risk factors, playing vital roles in various cardiovascular diseases such as calcific aortic valve disease (CAVD). We aim to explore the CKD-associated genes potentially involving CAVD pathogenesis, and to discover candidate biomarkers for the diagnosis of CKD with CAVD.

**Methods:**

Three CAVD, one CKD-PBMC and one CKD-Kidney datasets of expression profiles were obtained from the GEO database. Firstly, to detect CAVD key genes and CKD-associated secretory proteins, differentially expressed analysis and WGCNA were carried out. Protein-protein interaction (PPI), functional enrichment and cMAP analyses were employed to reveal CKD-related pathogenic genes and underlying mechanisms in CKD-related CAVD as well as the potential drugs for CAVD treatment. Then, machine learning algorithms including LASSO regression and random forest were adopted for screening candidate biomarkers and constructing diagnostic nomogram for predicting CKD-related CAVD. Moreover, ROC curve, calibration curve and decision curve analyses were applied to evaluate the diagnostic performance of nomogram. Finally, the CIBERSORT algorithm was used to explore immune cell infiltration in CAVD.

**Results:**

The integrated CAVD dataset identified 124 CAVD key genes by intersecting differential expression and WGCNA analyses. Totally 983 CKD-associated secretory proteins were screened by differential expression analysis of CKD-PBMC/Kidney datasets. PPI analysis identified two key modules containing 76 nodes, regarded as CKD-related pathogenic genes in CAVD, which were mostly enriched in inflammatory and immune regulation by enrichment analysis. The cMAP analysis exposed metyrapone as a more potential drug for CAVD treatment. 17 genes were overlapped between CAVD key genes and CKD-associated secretory proteins, and two hub genes were chosen as candidate biomarkers for developing nomogram with ideal diagnostic performance through machine learning. Furthermore, SLPI/MMP9 expression patterns were confirmed in our external cohort and the nomogram could serve as novel diagnosis models for distinguishing CAVD. Finally, immune cell infiltration results uncovered immune dysregulation in CAVD, and SLPI/MMP9 were significantly associated with invasive immune cells.

**Conclusions:**

We revealed the inflammatory-immune pathways underlying CKD-related CAVD, and developed SLPI/MMP9-based CAVD diagnostic nomogram, which offered novel insights into future serum-based diagnosis and therapeutic intervention of CKD with CAVD.

**Supplementary Information:**

The online version contains supplementary material available at 10.1186/s12967-023-04171-x.

## Introduction

Chronic kidney disease (CKD) is becoming a severe public-health concern, currently affecting over 8% population worldwide with the increasing incidence [1, 2]. A growing body of studies showed that CKD not only manifested as renal function decline but also featured as excessive mineral deposition, the inflammatory cascade and oxidative stress [3-6], all of which were strongly associated with the pathogenesis of various cardiovascular diseases, including atherosclerotic, myocardial infarction and aortic valvular cardiac disease [7]. Calcific aortic valve disease (CAVD) is the one of the most prevalent valvular diseases and is considered as the primary reason for aortic valve stenosis (AVS), which may eventually lead to devastating cardiac outcomes, such as severe heart failure and sudden cardiac death [8, 9]. Recent studies showed that CAVD was more commonly observed in CKD than in general populations, and CKD represents an independent risk factor for the prognosis of CAVD [3, 4], suggesting that CKD patients may exhibit a heightened risk of CAVD. Nevertheless, the underlying molecular mechanisms leading to CKD-related CAVD are complicated and obscure.

Increasing studies have proposed that excessive endogenous and exogenous mediators could induce sterile inflammation in CKD, releasing a variety of pro-inflammatory cytokines (e.g. IL-6, IL-1β and IL-18) which have been implicated in the progression of CKD and development of subsequent cardiovascular diseases [10]. Furthermore, CKD is characterized as pre-mature cellular senescence and displays a senescence-associated phenotype with the secretion of inflammatory mediators, Wnt/β-catenin signaling-related ligands [11] and TGF-β [12], leading to a cascade of ageing of the kidney and other targeted organs or tissues [13]. It should be noted that ageing is significantly involved in the pathological process of various diseases, especially in vascular calcification [14]. These studies suggest that CKD may contribute to subsequent complications including CAVD, at least partly through secretory proteins.

Over the past few decades, it has been widely acknowledged that CKD initiates and accelerates CAVD, which in turn increases the risk of death in CKD patients [15]. Therefore, early detection of CAVD in CKD patients is necessary, in order to conduct medical intervention before they develop clinical symptoms. As a result, it is urgent to develop a more comprehensive diagnostic model constructed with novel potential serum biomarkers for the early diagnosis of CAVD, especially of those in CKD patients, with high sensitivity and specificity.

In this study, we employed multiple integrative bioinformatics tools to reveal the hub genes and potential mechanism underlying CKD-related CAVD by collecting three CAVD datasets and two CKD datasets from the Gene Expression Omnibus (GEO) database. Potential compounds with therapeutic efficiency in CAVD were also identified. Furthermore, machine learning was carried out to construct a diagnostic nomogram model for CAVD prediction on the basis of the hub genes (SLPI and MMP9) that were discovered in CKD-related pathogenic genes. We validated the expression pattern of the hub genes and evaluated the diagnostic efficiency of the constructed nomogram in a small cohort of patients from our hospital. Finally, we explored the immune cells signatures of CAVD to uncover the association of the hub genes with the immunological landscape.

## Methods

### Microarray data collecting and processing

Three raw expression profile datasets of CAVD and control groups, including GSE12644, GSE51472 and GSE83453, were downloaded from the GEO database (https://www.ncbi.nlm.nih.gov/geo/) [16]. The microarray datasets of peripheral blood mononuclear cells (PBMC) (GSE37171) and kidney tissues (GSE66494) from CKD patients were also obtained from GEO as well. Detailed descriptive information of datasets was shown in Table 1. The integrated CAVD expression data was obtained by the batch correction of three CAVD datasets based on the combat function of “SVA” package [17] in R software (version 4.2.1), which finally contained 34 calcified samples and 23 control samples.

### Differentially expressed genes (DEGs) analysis

Background correction, normalization and gene symbol conversion were performed on the CAVD integrated dataset and CKD datasets (GSE37171 and GSE66494). Later, DEGs in CAVD and CKD datasets were identified using the “Limma” package [18] in R software. Therefore, DEGs in CAVD dataset were screened upon the thresholds of adjusted *p ≤ *0.05 and |log2 (fold change)| ≥ 1, whereas DEGs in CKD datasets were identified upon the thresholds of adjusted *p *≤ 0.05 and |log2 (fold change)| ≥ 0.585. Subsequently, the expression patterns of DEGs were visualized in the form of volcano plots and heatmaps with the “ggplot2” package and “pheatmap” package in R software, respectively.

### Weighted Gene Co-Expression Network Analysis (WGCNA) and key module genes identification

As a systematic biological approach, WGCNA was employed to reveal the gene association patterns among different samples and to detect the candidate biomarker genes or therapeutic targets according to the interconnectedness of gene sets together with the association between gene sets and phenotypes. As shown in Step1, the median absolute deviation (MAD) of each gene in the CAVD integrated dataset was calculated and then genes with MAD of 0 were removed from each sample. In Step2, the “goodSamplesGenes” function of the “WGCNA” package [19] was employed to examine the unqualified genes and samples. In Step3, the one-step network construction function of the “WGCNA” package was employed to construct a scale-free co-expression gene network. Meanwhile, the appropriate soft threshold power (β = 5) was taken as the weight value in this experiment. In Step4, after obtaining the modules, the different module eigengenes (ME) were obtained based on the first principal component of the module expression, while the module-trait relationships were evaluated in line with the association between MEs and clinical characteristics. In Step5, the modules with the most significant positive and negative correlations of module-trait relationships were screened. Then, MM and GS scores in modules were also evaluated to state the module significance (MS).

### Secretory proteins access

Secretory proteins were downloaded from The Human Protein Atlas database (https://www.proteinatlas.org/) [20]. A total of 3970 genes coding secretory proteins were downloaded from the protein class of “SPOCTOPUS predicted secreted proteins” (https://www.proteinatlas.org/search/protein_class%3ASPOCTOPUS+predicted+secreted+proteins).

### The construction of protein–protein interaction (PPI) network

To excavate the interactions between CKD-associated secretory proteins and the CAVD key genes, a PPI network linked with CKD and CAVD was established on the basis of the STRING database (https://www.string-db.org) [21], with a medium confidence score of > 0.4. Later, the PPI network was visualized by the Cytoscape software (version 3.8.2). Moreover, we further performed the Cytoscape plug-in molecular complex detection (MCODE) to detect the significant modules. Modules with top2 highest scores were chosen for performing further analysis.

### Functional enrichment analysis

To explore the biological function and concrete mechanism of the CKD-related pathogenic genes, we carried out Gene Ontology (GO) and Kyoto Encyclopedia of Genes and Genomes (KEGG) pathway enrichment analysis by importing the genes into the DAVID database (https://david.ncifcrf.gov/) [22]. A threshold of *p* < 0.05 was regarded to be significant enrichment. Additionally, the findings of functional enrichment analysis were displayed via bubble diagram and circos plot.

### Connectivity map (cMAP) analysis

CMAP (https://clue.io) [23], is a gene expression profile database based on the intervention of gene expression signatures, which can reveal relationships between diseases, genes, and small molecule compounds. In this study, upregulated genes from significant modules, which had the top2 highest scores identified in the CKD-CAVD PPI network, were incorporated into cMAP online database to discover the potential small-molecular drugs for CAVD treatment. Finally, the top10 compounds with highest enrichment scores were identified.

### Machine learning

To identify the candidate biomarkers and establish a diagnostic model of CAVD, the least absolute shrinkage and selection operator (LASSO) algorithm, a logistic regression method for filtering variables to enhance the predictive performance, was initially adopted in this work to screen the candidate biomarkers with the “glmnet” package [24]. Next, the random forest (RF) algorithm, integrating multiple trees through the idea of ensemble learning to gain better accuracy, was employed to narrow down the candidate biomarkers with the “randomForest” package [25] as well. The overlapping genes of LASSO model and the genes with the MeanDecreaseGini > 2 from RF model were defined as hub genes for developing a diagnostic model of CKD-related CAVD.

### The construction of nomogram and the assessment of diagnostic marker prediction model

The nomogram was constructed based on the two hub genes by using the “rms” package [26]. The area under the receiver operating characteristic (ROC) curve was drawn to evaluate the performance of each hub gene and the nomogram in the diagnosis of CAVD. Furthermore, ROC curve was performed to determine whether the nomogram-based decision was conducive to aortic valve sclerosis diagnosis. Finally, the calibration curves and decision curve analysis (DCA) were carried out in order to assess the nomogram predictive efficiency in CKD-related CAVD.

### Immune infiltration analysis

The “CIBERSORT” package [27] was executed to assess the number of the immune cell infiltration from the CAVD gene expression profile. The abundance and proportion of the immune infiltration were presented for each sample as barplot using the “ggplot2” package. The differences of the proportion of 22 types of immune cells between calcified and control aortic valve samples were compared by adopting Wilcoxon test, where *p* < 0.05 was regarded to be of statistical significance and was displayed by Stacked histogram based on the “ggplot2” package. Subsequently, the association of 22 types of invading immune cells was shown with the use of the “corrplot” package. Finally, Spearman’s rank correlation coefficient was adopted for the correlation analysis between the expression of diagnostic biomarkers and the content of infiltrated immune cells, and *p* < 0.05 was thought to be of statistical significance.

### Patients’ samples collection

Human calcified (n = 7) and non-calcified control (n = 5) aortic valve biopsies were obtained from the patients experiencing aortic valve replacement surgery from Sun Yat-sen Memorial Hospital of Sun Yat-sen University, Guangzhou, China. Moreover, human serum samples from healthy control individuals (n = 24), patients with CAVD (n = 24), and CKD patients (stage 3–5) with (n = 10) or without CAVD (n = 22), were also collected from Sun Yat-sen Memorial Hospital. Patients with congenital aortic valve abnormality, rheumatic disease, and endocarditis were excluded. The clinical information of patients was shown in Table 2. The protocols of human samples obtained approval from the Institutional Research Ethics Committee at Sun Yat-sen Memorial Hospital of Sun Yat-sen University.

**Table 1:** Descriptive statistics of the GEO datasets

GEO accession	Platform	Origin	Sample		Species
			Control	CAVD	
GSE12644	GPL570	Heart valve	10	10	Homo sapiens
GSE51472	GPL570	Heart valve	5	5	Homo sapiens
GSE83453	GPL10558	Heart valve	8	19	Homo sapiens

GEO accession	Platform	Origin	Sample		Species
			Control	CKD	
GSE37171	GPL570	PBMC	40	75	Homo sapiens
GSE66494	GPL6480	Kidney	5	47	Homo sapiens

**Table 2:** The clinical characteristics of patients from our cohort

Clinical variables	Control (n = 24)	CAVD (n = 24)	CKD (n = 22)	CKD with CAVD (n = 10)
Female/male (n)	11/13	12/12	6/16	3/7
Age	61 (48–74)	67 (57–77)	65 (56–74)	73 (62–84)
Serum creatinine (Scr, µmol/L)	71.36 ± 15.19	79.54 ± 13.57	533.93 ± 466.78	253.01 ± 344.48
Estimated glomerular filtration rate (eGFR, mL/min)	90.29 ± 17.44	78.14 ± 12.13	20.46 ± 19.43	43.50 ± 21.59

### The validation of the expression of hub genes between control and CAVD groups

Total RNA extraction was adopted using the Trizol reagent (Thermo Fisher Scientific, Darmstadt, Germany), followed by reverse transcription with a Reverse Transcription Kit (Ruizhen Bio, Guangzhou, China) following the instruction of the manufacturer. Real-time quantitative PCR (RT-qPCR) was performed by adopting a SYBR Green PCR Kit (Ruizhen Bio). All reactions were conducted in duplicate, and the relative mRNA expression was calculated based on the 2^−ΔΔCt^ approach. Primer sequences are listed as follows: SLPI-F, 5ʹ-GAGATGTTGTCCTGACACTTGTG-3ʹ; SLPI-R, 5ʹ-AGGCTTCCTCCT TGTTGGGT3ʹ; MMP9-F, 5ʹ-ACGCAGACATCGTCATCCAGT-3ʹ; MMP9-R, 5ʹ-G GACCACAACTCGTCATCGTC-3ʹ; GAPDH-F, 5ʹ-GAGTCAACGGATTTGGTCG T-3ʹ;  GAPDH-R, 5ʹ-GACAAGCTTCCCGTTCTCAG-3ʹ.

### The evaluation of diagnostic models in the external cohort

Serum samples were obtained from control individuals and CAVD patients as well as CKD patients with or without CAVD. In addition, the serum SLPI and MMP9 levels were determined with the indicated ELISA kits (Cusabio, Wuhan, China) in line with the manufacturer’s protocols.

### Statistical analysis

GraphPad Prism version 9.0.2 (GraphPad Software Inc., San Diego, CA, USA) was used for statistical analysis. Results were displayed as mean ± SD. Differences between the two groups were compared by unpaired Student′s t-test. *P* < 0.05 was regarded as statistical significance.

## Result

### Data processing

**Fig. 1:**
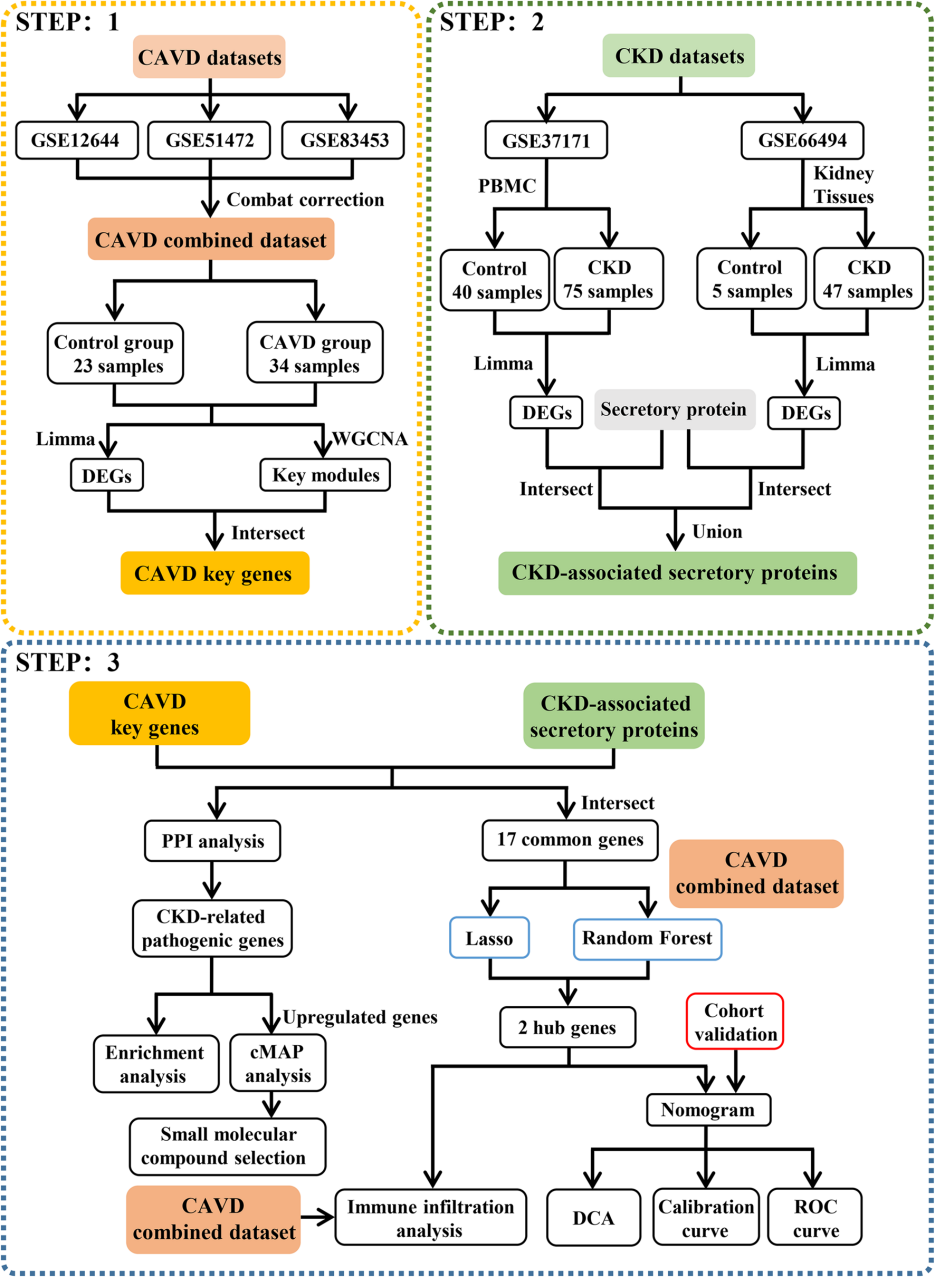
Flow chart of this study design

**Fig. 2:**
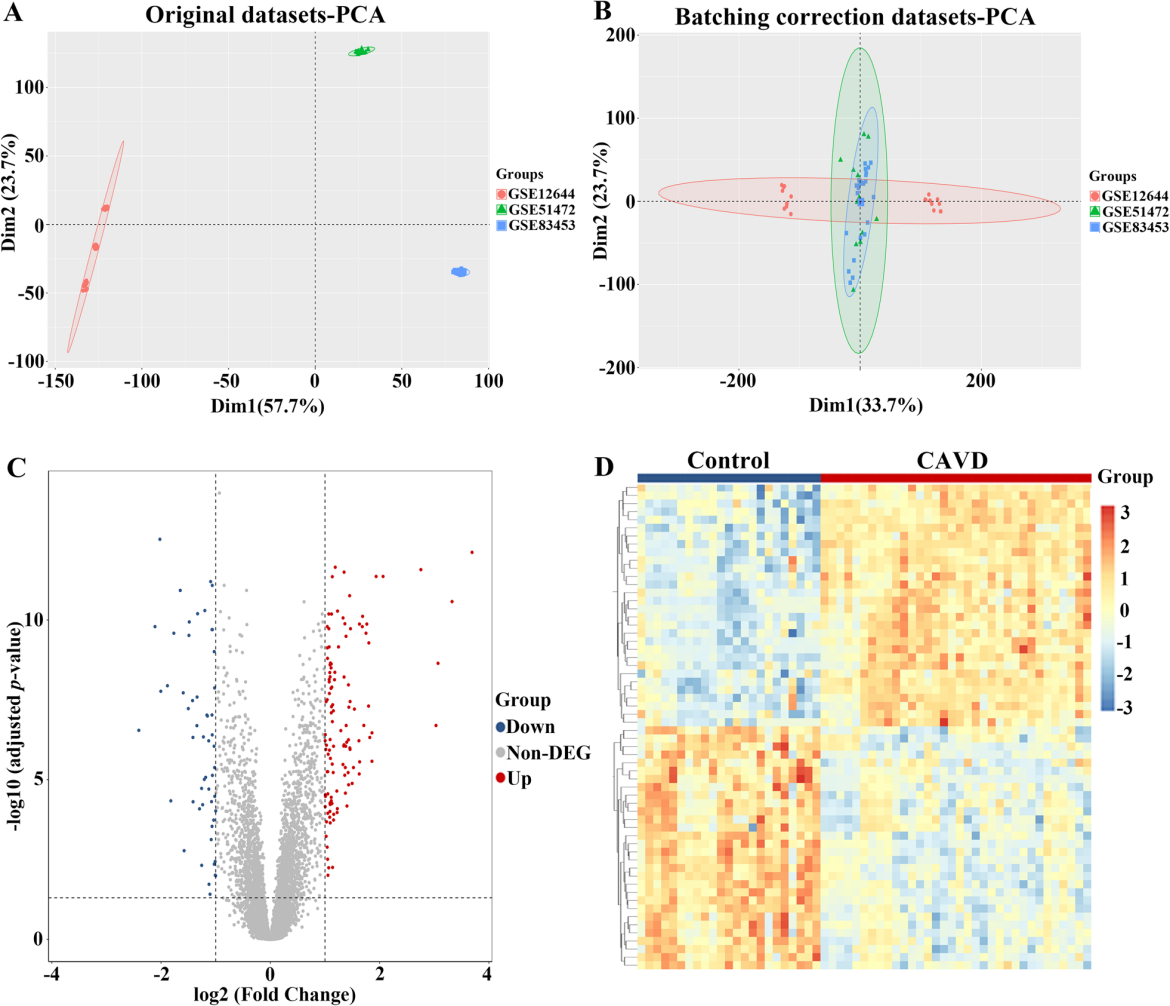
The integration of CAVD datasets and differential expression analysis of the integrated CAVD dataset. **A** PCA of three original CAVD datasets before batch-effect correction. **B** PCA of the integrated CAVD dataset after batch-effect correction. **C** The volcano plot representing CAVD DEGs in the integrated CAVD dataset. The upregulated genes are presented in red dots, whereas, the downregulated genes are presented in blue dots. **D** The heatmap showing the top 30 upregulated and 30 downregulated DEGs in the integrated CAVD dataset. *CAVD* calcific aortic valve disease, *PCA* principal component analysis, *DEGs* differentially expressed genes

The strategy of bioinformatics analysis is performed as shown in Fig. 1. Three raw datasets of calcified and control aortic valve samples were collected from the GEO database and combined after carrying out batch effect removal. After batch correction, the integrated CAVD dataset was obtained and normalized, including 34 calcified samples in the CAVD group and 23 control samples in the control group. As shown in Fig. 2A and B, the differences among three datasets were significantly decreased after batch effect removal.

### Identification of differentially expressed genes in calcific aortic valve disease

Differential analysis between combined calcified and control aortic valve samples revealed 173 differentially expressed genes (DEGs) with the cut-off criterion of adjusted *p* ≤ 0.05 and |log2 (fold change)| ≥ 1, containing 119 upregulated and 54 downregulated genes. Volcano plot and heatmap were applied to depict the expression pattern of DEGs in the integrated CAVD dataset (Fig. 2C and D).

### The construction of weighted gene co-expression network and the identification of key modules in CAVD

**Fig. 3:**
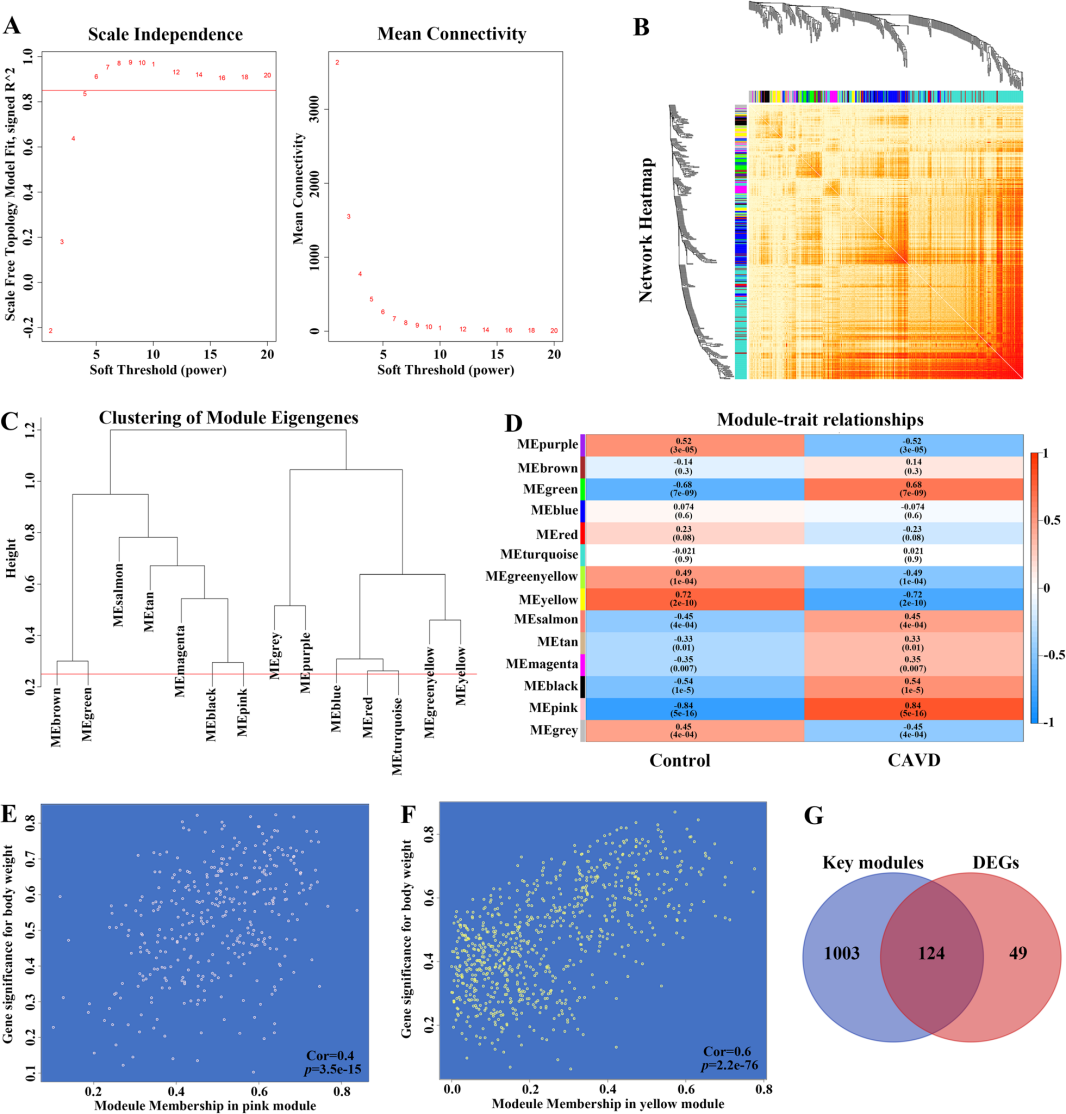
Screening of key module genes in the integrated CAVD dataset via WGCNA and identification of CAVD key genes through the intersection of key module genes and DEGs. **A** The scale-free topology model was utilized to identify the best β value, and β = 5 was chosen as the soft threshold based on the average connectivity and scale independence. **B** The network heatmap showing the gene dendrogram and module eigengenes. **C** The cluster dendrogram presenting module eigengenes. **D** The heatmap revealing the relationship between module eigengenes and status of CAVD. The correlation (upper) and *p*-value (bottom) of module eigengenes and status of CAVD were presented. The pink and yellow modules correlated with CAVD exhibited the highest and lowest correlation coefficients, respectively, which were identified as the key modules in CAVD. **E** The correlation plot between the pink module membership and the gene significance of genes in the pink module. **F** The correlation plot between the yellow module membership and the gene significance of genes in the yellow module. **G** A total of 124 key genes in CAVD were identified by taking the intersection between key modules genes and DEGs via the venn diagram. *WGCNA* weighted gene co-expression network analysis, *CAVD* calcific aortic valve disease, *DEG* differentially expressed genes

In order to further explore the key genes in CAVD, weighted gene co-expression network analysis (WGCNA) was carried out to identify the most relevant gene modules in calcified aortic valve samples. According to the scale independence and average connectivity, the soft-thresholding power of 5 was chosen (Fig. 3A). Totally 14 modules were generated using that power and the cluster dendrogram of the modules was presented in Fig. 3B. The clustering of module eigengenes was displayed in Fig. 3C. Furthermore, this study explored the correlation between CAVD and gene modules (Fig. 3D**)**. These data showed that the pink module exhibited the highest positive correlation with CAVD (358 genes, r = 0.84, *p* = 5e−16), whereas the yellow module displayed the most negative relation to CAVD (769 genes, r = − 0.72, *p* = 2e−10). On this basis, the pink and yellow modules were considered as the key modules for subsequent analysis. Moreover, we found a strong association between module membership and gene significance in the pink (r = 0.4, *p* = 3.5e−15) and yellow modules (r = 0.6, *p* = 2.2e−76), respectively (Fig. 3E, F**)**. Therefore, 1127 crucial genes that were significantly associated with CAVD were identified in the pink and yellow modules. In addition, we further intersected genes from DEGs and crucial genes from WGCNA in calcified aortic valve samples to identify the key genes in CAVD, obtaining totally 124 genes, which were further subjected to later analysis (Fig. 3G**)**.

### Identification of differentially expressed secretory proteins in chronic kidney disease

**Fig. 4:**
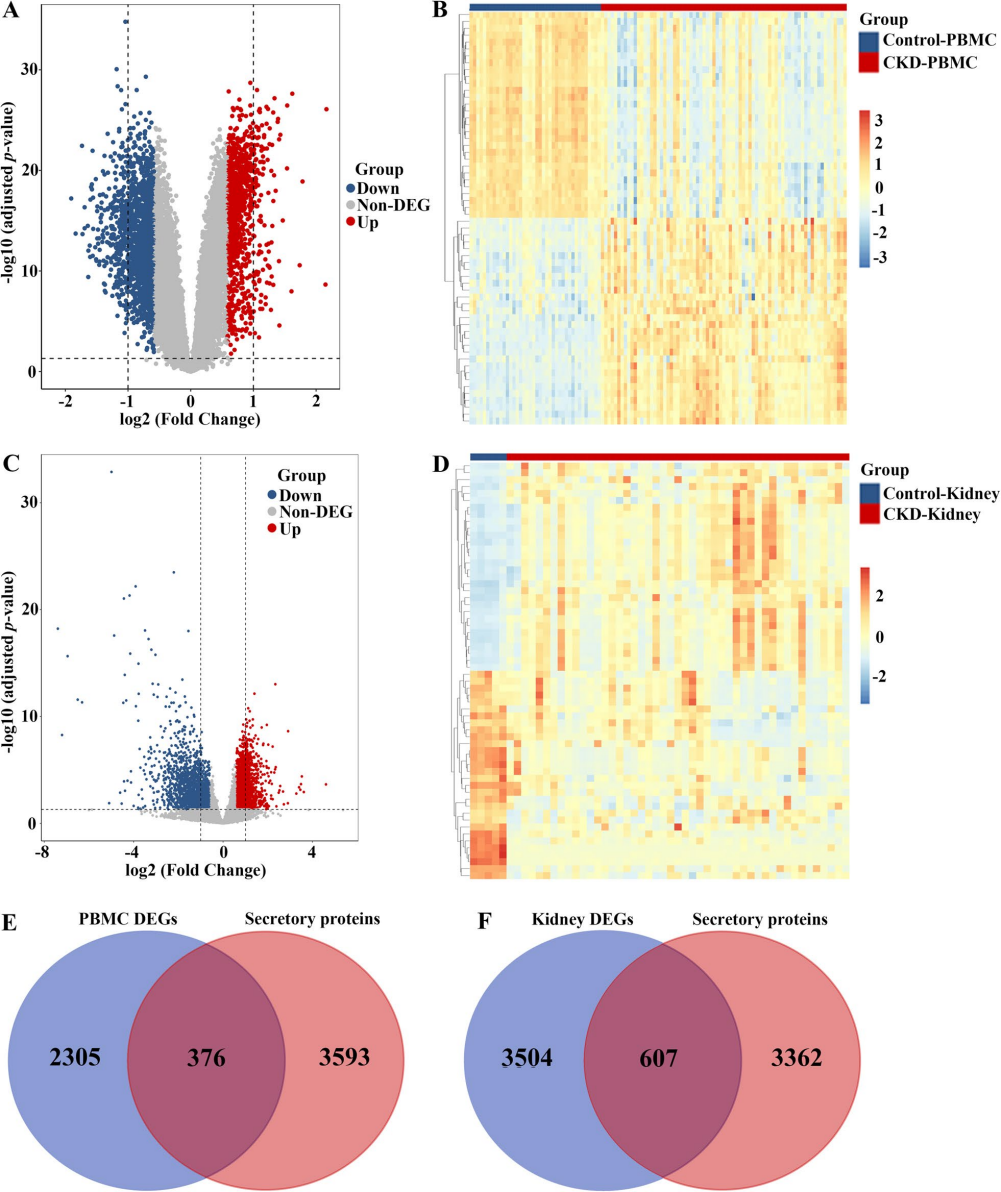
Identification of CKD-associated secretory proteins through differential expression analysis on secretory proteins in PMBC and kidney tissues of CKD. **A** The volcano plot revealing DEGs in the CKD-PMBC dataset. **B** The heatmap representing the top 30 upregulated and 30 downregulated DEGs in the CKD-PBMC dataset. **C** The volcano plot displaying DEGs in the CKD-Kidney dataset. **D** The heatmap displaying the top 30 upregulated and 30 downregulated DEGs in the CKD-Kidney dataset. **E** The intersection of CKD-PMBC DEGs with genes coding secretory proteins via the venn diagram. **F** The intersection of CKD-Kidney DEGs with genes coding secretory proteins via the venn diagram, and altogether 983 CKD-associated secretory proteins were identified. *CKD* chronic kidney disease, *PBMC* peripheral blood mononuclear cells, *DEG* differentially expressed genes

It is well known that CKD is causally linked to CAVD and possibly accelerates the occurence and progression of CAVD [15]. To investigate the pathogenic genes involved in CKD-related CAVD, we firstly re-analyzed the expression profiles of CKD peripheral blood mononuclear cell (PBMC) and CKD kidney tissues from the GEO database. As visualized via volcano plot and heatmap in Fig. 4A and D, totally 2681 DEGs were identified in CKD PBMC, while 4111 DEGs were discovered in CKD kidney tissues in line with the thresholds of adjusted *p* ≤ 0.05 and |log2 (fold change)|  ≥ 0.585. Considering that CKD may promote the onset and development of CAVD mainly by releasing secretory proteins, we then obtained the CKD-associated secretory proteins through the combination of 376 and 607 differentially expressed secretory proteins from CKD PBMC (Fig. 4E**)** and kidney tissues datasets (Fig. 4F**)**, respectively.

### Protein–protein interaction network and functional enrichment of the pathogenic genes involved in CKD-related CAVD

**Fig. 5:**
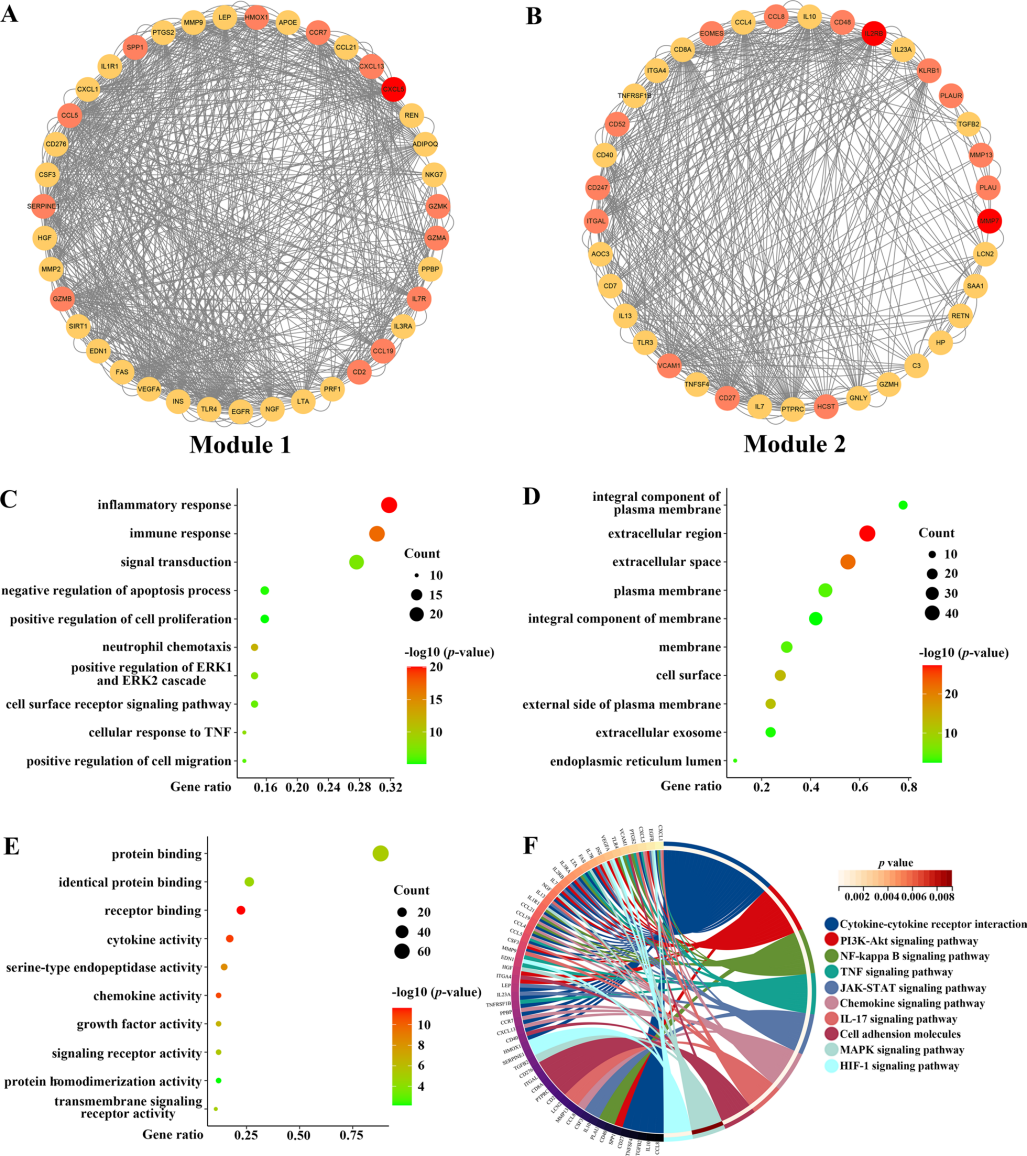
PPI analysis between CKD-associated secretory proteins and CAVD key genes, and followed by enrichment analysis of the PPI-screened nodes. **A** The PPI network of module1 genes with the top1 highest score based on Cytoscape plug-in MCODE analysis. Salmon nodes are marked as members of CAVD key genes, yellow nodes as members of CKD-associated secretory proteins, while red nodes as common genes of the two sets. **B** The PPI network of module2 genes with the top2 highest score according to MCODE analysis. **C**–**F** The bubble plots showing the GO enrichment analysis results, including biological process (**C**), cellular component (**D**), and molecular function (**E**) of genes included in module1 and module2. **F** Circos plot representing the KEGG analysis results of genes included in module1 and module2. *PPI* protein-protein interaction, *CKD* chronic kidney disease, *CAVD* calcific aortic valve disease, *MCODE* molecular complex detection

To reveal the potential pathogenic genes and underlying mechanism in CKD-related CAVD, the interaction of the CKD-associated secretory proteins and the key genes in CAVD was collected by the STRING database with a medium confidence score of > 0.4. The pathogenic genes in CKD-related CAVD were presented by the Cytoscape software and the top2 most significant modules were identified by adopting MCODE, in which the included 76 genes were identified as the CKD-related pathogenic genes. (Fig. 5A and B). To better understand the function and particular mechanism of the pathogenic genes, we imported the CKD-related pathogenic genes from the top2 significant modules into DAVID online database to perform functional enrichment and KEGG analysis. Biological process (BP) of Gene Ontology (GO) term analysis illustrated that the pathogenic genes in CKD-related CAVD were mostly enriched in “inflammatory response” and “immune response” (Fig. 5C). In terms of cellular component (CC) of GO term analysis, the pathogenic genes were mostly located in “integral component of membrane” and “extracellular region” (Fig. 5D). Concerning molecular function (MF) analysis, the results indicated that “protein binding” and “identical protein binding” were the most relevant items of the pathogenic genes (Fig. 5E). KEGG pathway analysis showed that the pathogenic genes in CKD-related CAVD were strongly associated with “cytokine-cytokine receptor interaction”, “PI3K-Akt signaling pathway” and “NF-Kappa B signaling pathway” (Fig. 5F).

### Identification of candidate small-molecular compounds for CAVD treatment

**Fig. 6:**
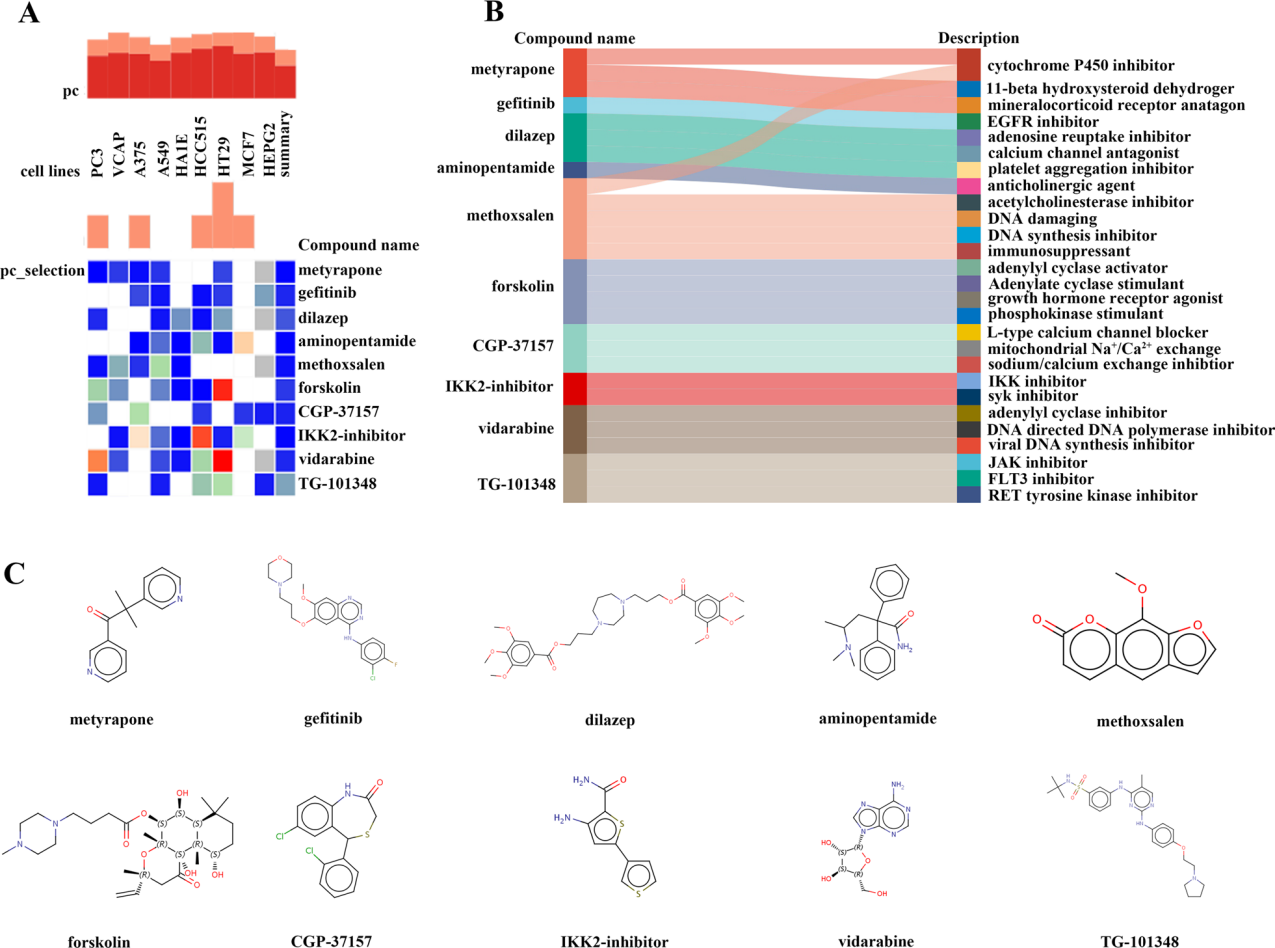
Screening of the potential small-molecular compounds for the treatment of CAVD via cMAP analysis. **A** The heatmap presenting the top10 compounds with the most significantly negative enrichment scores in 10 cell lines based on cMAP analysis. **B** The description of those top10 compounds. **C** The chemical structures of those 10 compounds were shown. *cMAP* connectivity map

To further investigate the potential small-molecular drugs that might exert a therapeutic effect in CKD-related CAVD patients, upregulated genes in calcified aortic valve samples from CKD-related pathogenic genes were imported into the connectivity map (cMAP) database to predict small-molecule compounds that could reverse the altered expression of CKD-related pathogenic genes in CAVD. Following the significant inquiry, the top10 compounds including metyrapone, gefitinib, dilazep, aminopentamide, methoxsalen, forskolin, CGP-37157, IKK2-inhibitor, vidarabine and TG-101348 with the highest negative scores were considered to be potential pharmacological therapeutic agents for the treatment of CKD-related CAVD (Fig. 6A). The description of the targeted pathways and chemical structures of these 10 compounds were displayed in Fig. 6B, C.

### Screening of hub genes harboring diagnostic value via machine learning and construction of a diagnostic model in CKD-related CAVD

**Fig. 7:**
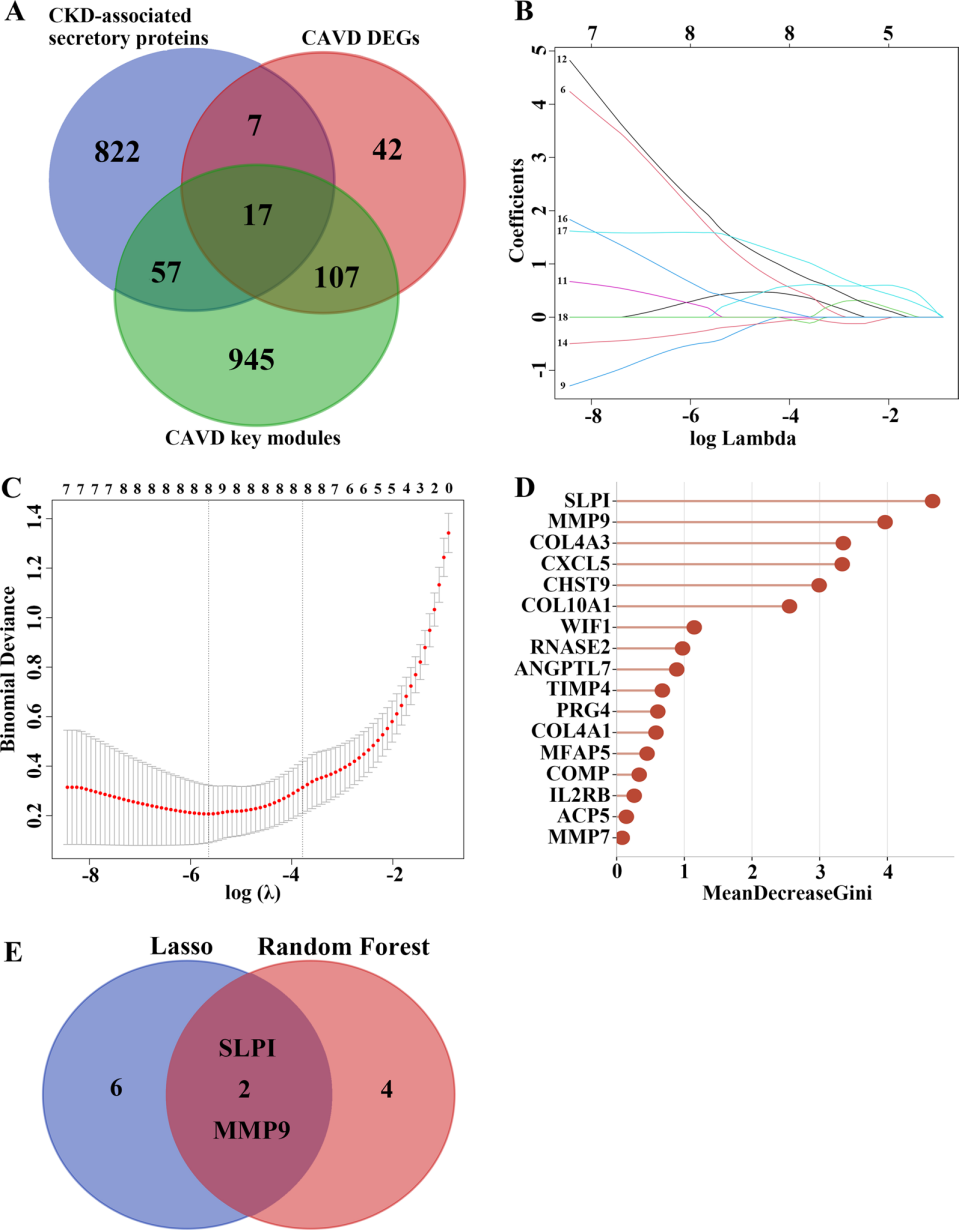
Identification of potential diagnostic biomarkers for CKD-related CAVD by the machine learning methods. **A** The venn diagram showing the 17 overlapping genes of CKD-associated secretory proteins, CAVD DEGs and CAVD key modules genes. **B**, **C** The minimum (**B**) and lambda values (**C**) of diagnostic biomarkers (n = 8) were identified by the LASSO logistic regression algorithm. **D** The RF algorithm presenting the MeanDecreaseGini of the 17 genes in CAVD and 6 biomarkers with the score more than 2.0 were selected. **E** The venn diagram displaying two common genes between LASSO and RF algorithms, which were identified as the hub genes in CKD-related CAVD. *CKD* chronic kidney disease, *CAVD* calcific aortic valve disease, *DEGs* differentially expressed genes, *LASSO* least absolute shrinkage and selection operator, *RF* random forest

**Fig. 8:**
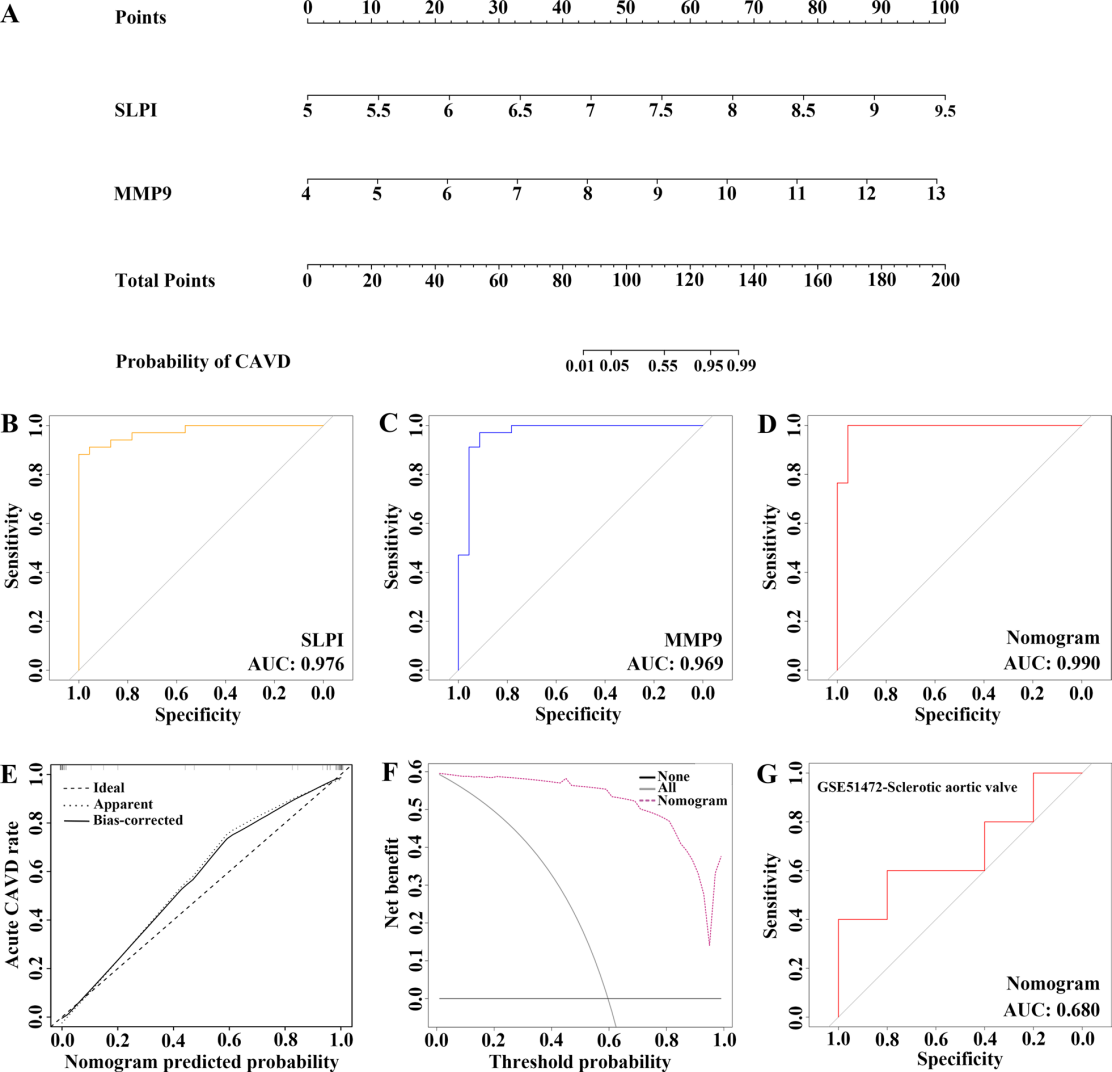
Development of the diagnostic nomogram model and efficacy assessment. **A** The nomogram was constructed based on the diagnostic biomarkers. **B–D** The ROC curve for the diagnostic performance of each candidate biomarker including SLPI (**B**), and MMP9 (**C**) and the nomogram model (**D**) constructed for CKD-related CAVD. **E** The calibration curve of nomogram model prediction in CKD-related CAVD. The dash line is marked as “Ideal”, which represents the standard curve, and is on behalf of the perfect prediction of the ideal model. The dotted line is marked as “Apparent”, which indicates the uncalibrated prediction curve, while the solid line is marked as “Bias-corrected” and represents the calibrated prediction curve. **F** DCA for the nomogram model. The black line is marked as “None”, which stands for the net benefit of the assumption that no patients have CAVD. The grey line is marked as “All”, which indicates the net benefit of the assumption that all patients have CAVD, and the purple line is marked as “Nomogram”, and represents the net benefit of the assumption that CKD-related CAVD are identified according to the diagnostic value of CAVD predicted by the nomogram model. **G** The ROC curve for the diagnostic performance of our nomogram model in predicting patients with sclerotic aortic valve from the GEO database. *ROC* receiver operating characteristic, *DCA* decision curve analysis, *CAVD* calcific aortic valve disease

Since the common differentially expressed secretory proteins between CAVD and CKD may play critical roles in CKD-related CAVD patients, 17 common genes were identified in the junction of CKD-associated secretory proteins and the key genes in CAVD, and they were subjected to subsequent construction of a CAVD diagnostic model which might distinguish CKD patients with or without CAVD (Fig. 7A). The LASSO regression algorithm was applied to identify eight potential candidate genes out of 17 common genes with a great effect on diagnosing CKD-related CAVD patients (Fig. 7B, C). To further narrow down the diagnostic biomarkers, Random Forest (RF) machine learning algorithm was also carried out to rank the 17 common genes in the lights of the variable importance of each gene, and the genes with the MeanDecreaseGini > 2 were extracted (Fig. 7D). Interestingly, after superposing the eight candidate genes from LASSO and six potential genes from RF, only two hub genes were overlapped in both subsets, containing secretory leukocyte protease inhibitor (SLPI) and matrix metalloproteinase 9 (MMP9) (Fig. 7E). For the better performance in diagnosis and prediction, nomogram was constructed on the basis of the two hub genes by performing logistics regression analysis (Fig. 8A). The receiver operating characteristic (ROC) curve was applied to evaluate the area under the curve (AUC) values of each hub gene and nomogram to determine their sensitivity and specificity for the diagnostic efficacy of CKD-related CAVD. As we expected, both two hub genes displayed AUC values > 0.9 and nomogram presented a higher AUC value than each hub gene, suggesting that nomogram may have a strong diagnostic value for CKD-related CAVD (Fig. 8B–D). The calibration curves uncovered that the predicted probability of the constructed nomogram diagnostic model was almost identical to that of the ideal model (Fig. 8E). Moreover, the DCA for the nomogram was also performed, showing that decision-making according to the nomogram model may be beneficial for the diagnosis of CKD-related CAVD (Fig. 8F). Sclerosis was the early stage of CAVD. The nomogram also demonstrated an ideal predictive value among CKD patients with sclerotic aortic valve in the GSE51472 dataset of the GEO database, which included 5 samples of human sclerotic aortic valve tissues and 5 samples of human normal aortic valve tissues (Fig. 8G), implying that the nomogram model could exhibit good diagnostic efficacy for early CAVD patients with CKD as well.

### Immune cell infiltration and correlation analysis of hub genes with invading immune cells in CAVD

**Fig. 9:**
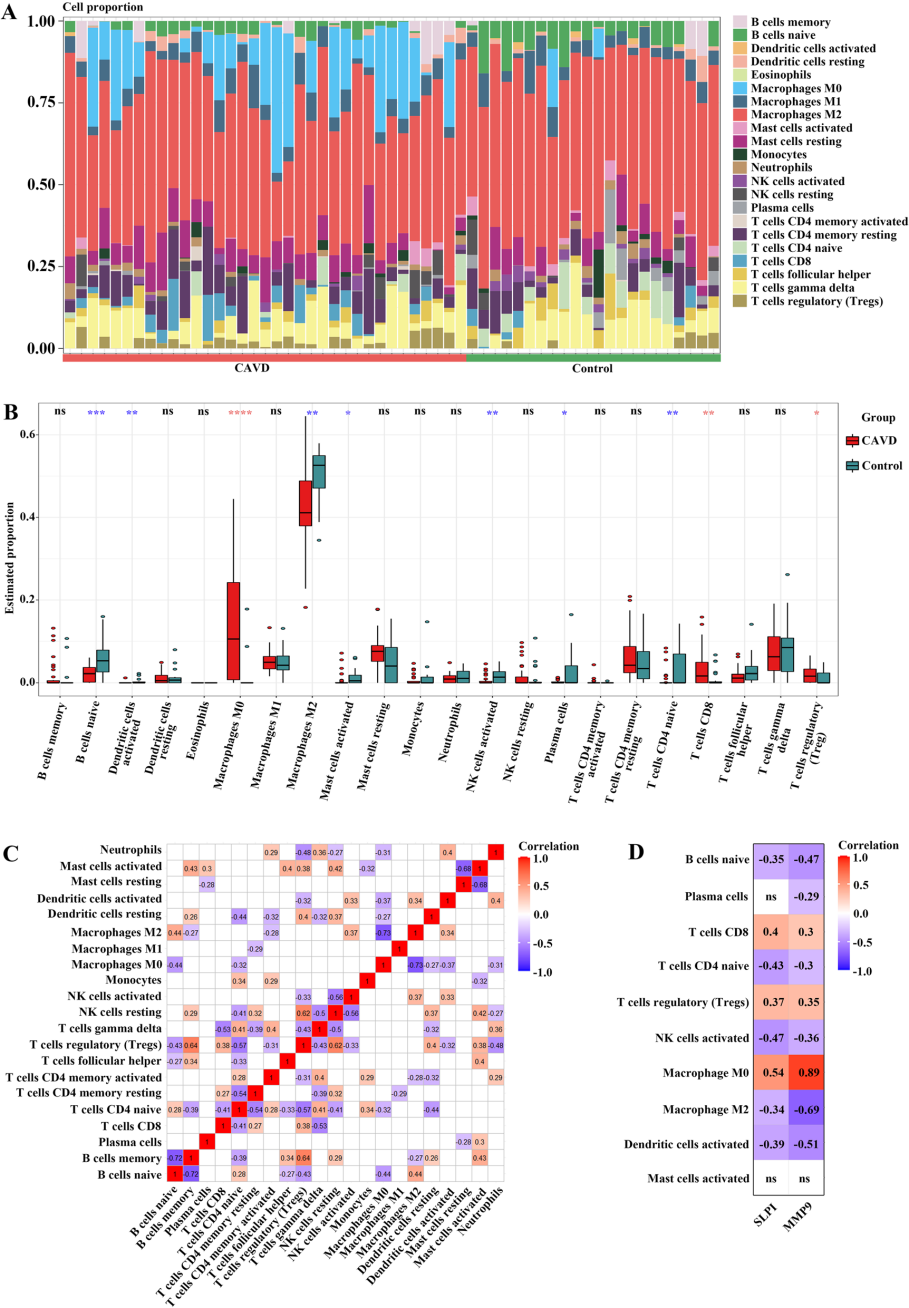
Immune cell infiltration analysis in CAVD. **A** Stacked histogram displaying the immune cell proportions between CAVD and control groups. **B** Violin plot showing the comparison of 22 kinds of immune cells between CAVD and control groups. Red and blue stars represent the increased and decreased proportions of immune cells in CAVD group, respectively. **C** The heatmap revealing the correlation of 22 kinds of immune cells infiltration upon the threshold of *p* < 0.05. **D** The correlation map representing the association of the differentially infiltrated immune cells with two hub genes upon the threshold of *p* < 0.05. *CAVD* calcific aortic valve disease. * *p* < 0.05; ** *p* < 0.01; *** *p* < 0.001; **** *p* < 0.0001; *ns* not significant

We found that the function and pathway analysis of CKD-associated pathogenic genes in CAVD showed a close association with inflammatory and immune processes. The CIBERSORT algorithm was performed to derive the characteristics of immune cells and explore the immune regulation as well as the correlation of diagnostic biomarkers with immune cell infiltration in CAVD. Figure 9A revealed the proportion of 22 types of immune cells in each sample, and significant differences were obtained between calcified and control aortic valve samples in 10 immune cell subpopulations. Compared with control group, CAVD displayed higher proportions of Macrophages M0, T cells CD8 and T cells regulatory (Tregs), whereas lower proportions of B cells naive, Dendritic cells activated, Macrophages M2, Mast cells activated, NK cells activated, Plasma cells and T cells CD4 naive (Fig. 9B). In addition, the correlation analysis of 22 types of immune cells indicated that T cells CD4 naive showed significantly positive correlation to Tregs (r = 0.57, *p* < 0.05), and that Mast cells activated were negatively associated with Dendritic cells activated (r = − 0.68, *p* < 0.05) (Fig. 9C). Moreover, the association between the expression of two hub genes and the proportion of differentially infiltrated immune cell types was further explored. As displayed in Fig. 9D, the hub genes, SLPI and MMP9, both demonstrated significant correlation to immune cell accumulation in CAVD.

### The validation of the expression pattern of two hub genes and the evaluation of the diagnostic value of the nomogram models

**Fig. 10:**
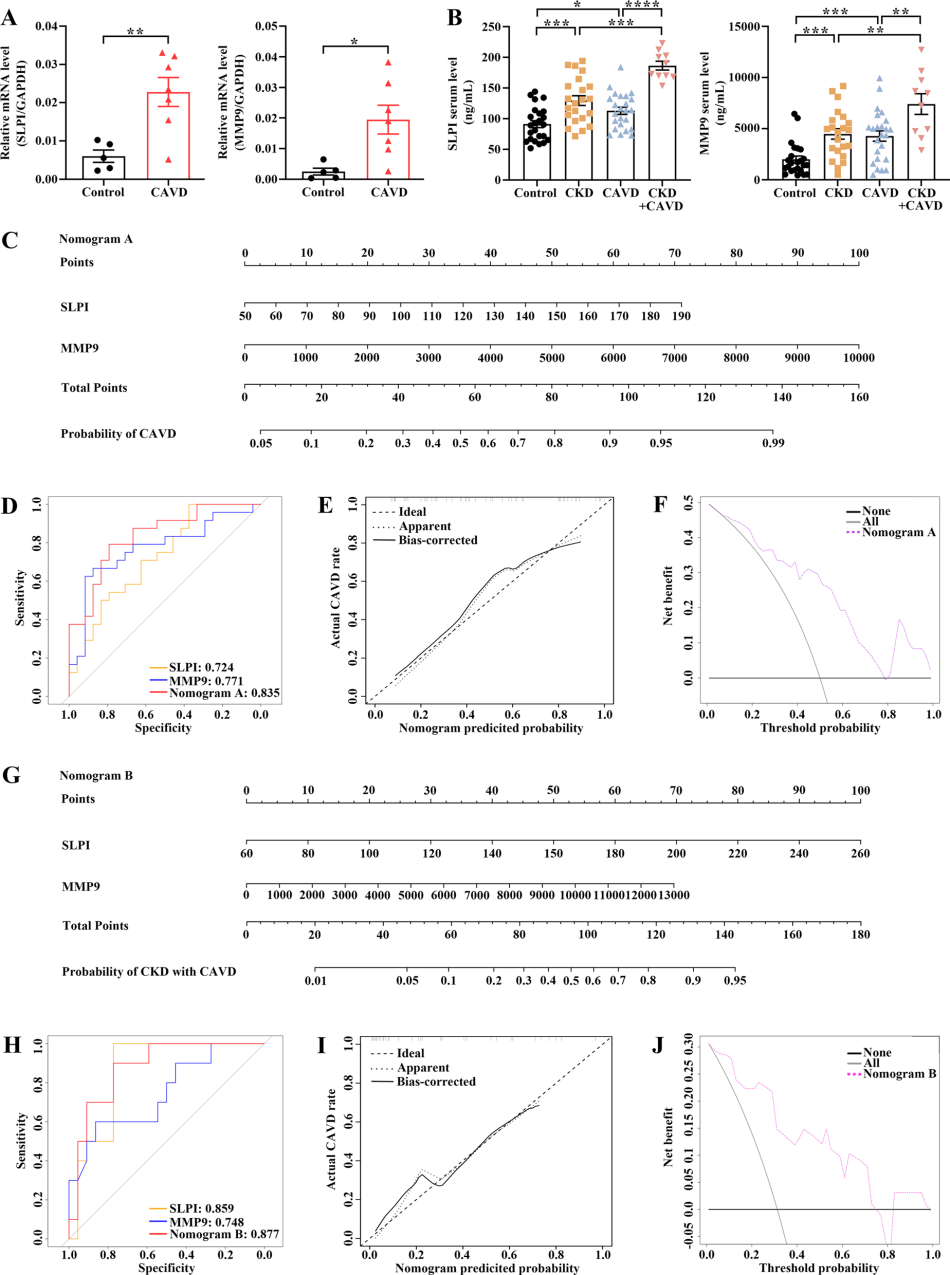
Validation of the expression patterns of two hub genes in calcified aortic valve samples and evaluation of the diagnostic performance of nomogram models in distinguishing CAVD. **A** RT-qPCR showing increased mRNA levels of SLPI and MMP9 in calcified aortic valve samples. **B** ELISA analysis displaying elevated serum SLPI and MMP9 levels in CKD and CAVD patients as well as CKD patients with CAVD. **C** The nomogram A was developed based on the diagnostic biomarkers to predict the risk of CAVD. **D** The ROC curves for the predictive performance of each candidate biomarker (SLPI and MMP9) and nomogram A. **E** The calibration curve of nomogram A prediction in CAVD patients. **F** DCA for the nomogram A. **G** The nomogram B was developed based on the diagnostic biomarkers to predict the risk of CKD patients with CAVD. **H** The ROC curves for the predictive performance of each candidate biomarker (SLPI and MMP9) and nomogram B. **I** The calibration curve of nomogram B prediction in CKD patients with CAVD. **J** DCA for the nomogram B

To further confirm the accuracy of the above integrated bioinformatics analysis, we firstly examined the expression pattern of the two hub genes in the recruited patients from our external cohort. The RT-qPCR results confirmed consistent upregulated expression pattern of two hub genes in calcified aortic valve samples in comparison with control aortic valve samples (Fig. 10A). Moreover, SLPI and MMP9 could be detected in the serum by ELISA and the levels were significantly elevated in CKD and CAVD patients as well as CKD patients with CAVD (Fig. 10B). Then, we developed a CAVD diagnostic nomogram model (named nomogram A) based on our cohort to predict the possibility of CAVD from control and CAVD groups (Fig. 10C). According to the ROC curves, the highest AUC of nomogram A could be observed between control and CAVD patients when compared to that of each biomarker (Fig. 10D). In addition, the calibration curves and DCA for assessing nomogram A showed that decision-making based on the nomogram A may favor the prediction of CAVD (Fig. 10E, F). Furthermore, another diagnostic nomogram model (named nomogram B) was also constructed to distinguish CKD patients with or without CAVD (Fig. 10G). Similarly, ROC and calibration curves as well as DCA indicated ideal predictive value of nomogram B for the CKD patients with CAVD (Fig. 10H–J**)**.

## Discussion

In recent years, with the widespread applications of microarray and sequencing methods, the molecular landscape and potential mechanisms of miscellaneous diseases can be easily explored [28, 29]. In addition, integrative bioinformatics analysis and machine learning tools are increasingly performed to explore the novel genes, potential diagnostic/prognostic biomarkers, underlying mechanisms, and prospective therapeutic targets based on the big data, which can shed more lights on the diseases [30, 31].

By applying a variety of comprehensive bioinformatics analysis approaches, to our knowledge, the present study is the first to excavate CKD-related pathogenic genes to elucidate the association between CKD and subsequent CAVD. It was surmised that inflammatory and immune processes together with signaling pathways including “cytokine-cytokine receptor interaction”, “PI3K-Akt signaling pathway” and “NF-Kappa B signaling pathway” might be the potential mechanisms underlying CKD-related CAVD. Moreover, two immune-related hub genes, SLPI and MMP9, were employed to develop diagnostic nomogram models to predict the risk of CAVD by machine learning approaches. According to our results, these two hub genes displayed ideal predictive performance for CAVD, as assessed by the ROC curve. At last, through the external validation of our cohort, the upregulated expression patterns of SLPI and MMP9 were confirmed to be consistent with the obtained datasets, and the diagnostic nomogram models based on SLPI and MMP9 levels performed well in significantly differentiating CAVD, particularly CAVD in CKD patients.

Increasing clinical studies have suggested that patients with CKD suffer a significantly increased incidence [32] and accelerated progression of CAVD [33]. As speculated in previous studies, CKD contributes to vascular calcification through calcium deposition, hyperphosphatemia and reactive oxygen species (ROS). Additionally, CKD is assumed to play a significant role in cardiovascular diseases partially by means of excreting secretory proteins, such as pro-inflammatory cytokines, TGF-β and bone-related proteins [34]. However, the potential factors and mechanisms participating in CKD-related CAVD are not fully understood.

CAVD is previously considered as a degenerative disease that occurs with age, however, a growing amount of evidence starts to realize that CAVD is an active pathological change, which is driven by a series of proactive multifactorial processes, including cellular transformation, apoptosis, oxidative stress and immune response [35]. Lately, the roles of inflammation and immunoregulation in the pathogenesis of CAVD have aroused an increasing attention. According to a previous report, the number of leukocytes in the aortic valve increases from 5% at birth to about 12% at 60 days of age [36]. Besides, local macrophages, CD4+ and CD8+ T lymphocytes are found to be activated in the calcified valve, leading to the production of more proinflammatory factors [37]. Furthermore, valvular osteoblast differentiation of valvular interstitial cells (VICs) may be promoted by invading monocytes and macrophages, at the same time, these cells themselves undergo calcification via secreting tumor necrosis factor (TNF) [38]. In this study, the GO-biological process annotation and KEGG enrichment analyses showed that the CKD-related pathogenic genes for CAVD were mostly enriched in the inflammatory and immunological relevant pathways, indicating that the inflammatory-immune pathways might be the potential mechanism in CKD-related CAVD.

Currently, the effective pharmacotherapy for the treatment of CAVD is still lacking, in this regard, it is urgently needed to explore the potential drugs. Numerous important breakthroughs have been made in the past few years in identifying small-molecular compounds with therapeutic potential in a variety of diseases. Small-molecular compounds exhibit several advantages, including high tissue penetration, a tunable half-life and oral bioavailability, making them more effective on treating patients [39]. Quinazoline-4-piperidine sulfamides (QPS) have been depicted as the inhibitors of Ectonucleotide pyrophosphatase/PDE1 (NPP1), which can attenuate the high phosphate-induced mineralization in a cellular model of CAVD [40]. However, no previous studies have disclosed potential small-molecular compounds for therapeutic application of CAVD based on gene expression signatures in the calcified aortic valve via high-throughput screening. Herein, by cMAP analysis, this study provided a novel perspective linking CKD-related pathogenic genes to discover the potential compounds targeting CAVD. The upregulated CKD-related pathogenic genes in the calcified valve were applied to cMAP analysis, and 10 small-molecular compounds (metyrapone, gefitinib, dilazep, aminopentamide, methoxsalen, forskolin, CGP-37157, IKK2-inhibitor, vidarabine and TG-101348) were selected as candidates. Of note, metyrapone, a potent inhibitor of 11-beta hydroxysteriod dehydroger and mineralocorticoid receptor as well as cytochrome P450, showed the highest negative enrichment score in cMAP analysis, implying that it maximally reversed the expression of upregulated CKD-related pathogenic genes in CAVD. Although no direct link is found between metyrapone and calcification, increasing studies have reported that metyrapone can ameliorate numerous cardiovascular disease such as cardiac remodeling [41] and endothelial dysfunction [42] by abrogating corticosterone signaling. Interestingly, the previous studies have established the pathogenic roles of CKD-related corticosterone signaling in vascular calcification [43, 44]. In addition, the metyrapone-mediated corticosterone inhibition also suppresses the production of pro-inflammatory factors, expression of adhesive molecules and accumulation of monocytes in neurovascular disorder [45]. On the basis of the above previous findings, the therapeutic effects of metyrapone make it possible to be a potential agent for the treatment of inflammatory and immunological diseases including CAVD. Thus, it is speculated that early medical intervention with metyrapone in CKD patients may not only improve the kidney function but also inhibit the initiation and progression of CAVD, finally significantly prolong the life span of patients.

Over the past few decades of life, CAVD is usually asymptomatic, but once symptoms occur, CAVD has often stepped into the severe stage. In this case, aortic valve replacements, either by surgical or transcatheter approach, are the only effective treatments, which are associated with the disadvantages of high costs and a high complication rate. Consequently, it is beneficial to diagnose and prevent CAVD in the early stage. It is estimated that one third of the aged population are diagnosed with the early stage of CAVD features, as indicated by the echocardiographic or radiological evidence [46]. Limited by the skills of the echocardiography operator and the quality of the imaging, it is needed to identify more conventional serum biomarkers for the early diagnosis of CKD patients with CAVD. Most noteworthily, a more comprehensive diagnostic nomogram model was established based on two hub genes in this study, which presented a higher diagnostic value for CKD-related CAVD than that of an independent biomarker. Moreover, the nomogram model was efficient in diagnosing patients with sclerotic aortic valve, indicating that this diagnostic nomogram was also potent in predicting the early stage of CAVD. Furthermore, external validation from our cohort revealed the elevated SLPI and MMP9 mRNA levels in aortic valve tissues of CAVD groups compared with control groups. Serum SLPI and MMP9 levels were also increased in patients with CAVD and higher in patients with CAVD and CKD, and our constructed diagnosis nomogram was capable of significantly distinguishing CAVD as well as CAVD in CKD patients.

SLPI belongs to the family of whey acidic proteins [47], which plays an important role in inhibiting human neutrophil-derived serine proteases, such as elastase and cathepsin G [48, 49]. Previous evidence suggests that SLPI may be a novel biomarker and target candidate for acute kidney injury (AKI), indicated by upregulation of SLPI mRNA levels in AKI allografts as well as elevated protein levels of SLPI in plasma and urine of AKI patients [50]. Moreover, it was identified as a novel biomarker for CKD patients with CAVD in our study. SLPI is principally expressed in epithelial cells, but it can also be secreted by endothelial cells, adipocytes and host-defense effector cells [49, 51, 52]. SLPI has been extensively reported to exert its function via several significant biological processes, such as host defense, inflammatory response and cell fate regulation [48]. Upregulation of SLPI increases the levels of osteoblast-related markers including Runx2, Sp7 and Col1a1 in MC3T3-E1 cells (the mouse osteoblast cell line), and promotes the proliferation of MC3T3-E1 cells [53]. Therefore, SLPI activation can strengthen osteoblast differentiation and proliferation. Noteworthily, aortic VIC undergoing osteoblast differentiation is found to have a critical effect on the development process and promote the progression of CAVD [54, 55]. However, the mechanisms regarding of SLPI in CAVD have not been elucidated yet. In this study, SLPI, as an important regulatory factor for inflammation and immunology, showed increased expression in calcified aortic valve in comparison with control aortic valve samples. In this regard, our study indicated that SLPI might provide a potential diagnostic indicator for CKD patients with CAVD.

Besides, MMP9 was identified as the perspective contributor to the diagnosis of CKD patients with CAVD in this study. MMP9, which belongs to the zinc-dependent endopeptidase family, is involved in immunology activation, inflammatory cascade regulation, extracellular matrix (ECM) disassembly and remolding to afford ways for immune cell accumulation in the pathogenesis of different diseases. A few studies have indicated that MMP9 contributes to atherogenesis through facilitating the migration of vascular smooth muscle cells and the invasion of macrophages. In addition, arterial stiffening is credited with the elevated expression of MMP9 since it plays a certain role in elastin degradation, leading to subsequent matrix remodeling. An earlier study has identified MMP9 as a pathogenetic factor for calcified aortic valve stenosis, and inhibition of MMP9 attenuates reactive oxygen species production and calcium deposition by improving the mitochondrial morphology and metabolism in calcified aortic valve interstitial cells [56]. Furthermore, the increased levels of circulating MMP9 is significantly associated with diabetic nephrology progression, and is specially involved in the development of albuminuria in patients with CKD [57]. Interestingly, our data suggested that the expression of MMP9 was significantly upregulated in CKD patients with CAVD. As a result, it was speculated that MMP9 might interrupt the balance between the anabolism and catabolism of ECM, and promote macrophage infiltration to participate in CAVD progression. Conclusively, MMP9 is assumed to be an appropriate biomarker for distinguishing calcification.

In immune cell infiltration analysis, the accumulation of various types of immune cells has been demonstrated to exist in all stages of CAVD, which is significantly related to the severity of aortic stenosis [58-60]. Previous studies have demonstrated that calcified aortic valve tissues and peripheral blood harbor diverse kinds of activated T lymphocytes [61, 62], where T cells CD8 exhibit a greater invasion ability than other subpopulations [62]. Moreover, activated T cells CD8 contribute to CAVD via secreting IFN-γ, eventually facilitating the progression of aortic stenosis [63]. Furthermore, macrophages, the heterogeneous innate immune system cells, can be classified as two major phenotypes, including pro-inflammatory M1 macrophages and anti-inflammatory M2 macrophages [64]. They can modulate phenotypic switch rapidly in response to the local microenvironment. Both M1 and M2 macrophages are reported to be accumulated in patients with CKD, with a lower proportion of M2 macrophages being detected in calcified aortic valves. In this study, significant differences in the infiltration of immune cells were identified between CAVD and control groups, with higher abundances of Macrophages M0, T cells CD8 and Tregs, whereas lower proportions of B cells naive, Dendritic cells activated, Macrophages M2, Mast cells activated, NK cells activated, Plasma cells and T cells CD4 naive. Furthermore, the hub genes SLPI and MMP9 showed close association with immune cell infiltration in CAVD, implying that the candidate biomarkers might not only distinguish CAVD but also contribute to CAVD by interaction with inflammatory-immune pathways. Thus, it is vital to comprehensively understand the inflammatory-immune pathways related to CAVD in order to develop novel diagnostic or prognostic biomarkers and therapeutic targets for CAVD.

## Supplementary Information

**Additional file 1.** All bioinformatics analysis codes.

## Data Availability

The public datasets were downloaded and analyzed in this study, which can be found in GEO data repository and included the accession numbers as follows: GSE12644, GSE51472, GSE83453, GSE37171, GSE66494, and GSE51472. All used source codes of bioinformatics analysis are shown in the Additional file 1.
