# Supplementary material for: Screening of immune-related secretory proteins linking chronic kidney disease with calcific aortic valve disease based on comprehensive bioinformatics analysis and machine learning
Source: J Transl Med. 2023 Jun 1;21:359. doi: 10.1186/s12967-023-04171-x (PMC10234004; doi:10.1186/s12967-023-04171-x)
Supplement: Supplementary file 1 — Additional file 1. All bioinformatics analysis codes. [file 12967_2023_4171_MOESM1_ESM.docx]

**Figure 2**

**Batch correlation working sheets:**

library("sva")

setwd("")

GSE12644 <- read.table("GSE12644/GSE12644_normalization_log2_symbol.txt",header=T,row.names=1,sep="\t")

GSE51472<-read.table("GSE51472/GSE51472_normalization_log2_symbol.txt",header=T,row.names=1,sep="\t")

GSE83453 <- read.table("GSE83453/GSE83453_normalization_log2_symbol.txt",header=T,row.names=1,sep="\t")

mrna_names <- intersect(rownames(GSE12644),rownames(GSE51472))

mrna_intersect <- intersect(mrna_names,rownames(GSE83453))

expr <- cbind(GSE12644[mrna_intersect,],GSE51472[mrna_intersect,],GSE83453[mrna_intersect,])

write.table(expr,"result_3sets/mrna_nocombat_3sets.txt",sep="\t")

batch <- paste0("batch",rep(c(1,2,3),c(20,10,27)))

tissue <- rep(c("Control","AVD","Control","AVD","AVD","Control"),c(10,10,5,5,19,8))

table(batch,tissue)

mod <- model.matrix(~tissue)

expr_new <- na.omit(expr) #去除NA

expr_batch<-ComBat(dat=expr_new,batch=batch,mod=mod)

write.table(expr_batch,"result_3sets/mrna_expr_3sets_batch.txt",sep="\t")

write.table(expr_batch[,1:20],"result_3sets/GSE12644_after_batch.txt",sep="\t")

write.table(expr_batch[,21:30],"result_3sets/GSE51472_after_batch.txt",sep="\t")

write.table(expr_batch[,31:57],"result_3sets/GSE83453_after_batch.txt",sep="\t")

**differential expression analysis:**

library(limma)

setwd("...")

GSE_expr <- read.csv(file ="result_3sets", header =TRUE, sep="\t", row.names=1,stringsAsFactors = FALSE, check.names = FALSE)

phenotype <- read.csv(file = "phenotarget.txt", header =TRUE, sep="\t", row.names=1,stringsAsFactors = FALSE, check.names = FALSE)

Group <- factor(phenotype$Group,levels = c("Control","AVD"))

design = model.matrix(~0+Group)

colnames(design) <- c('Control','AVD')

fit <- lmFit(GSE_expr, design)

contrast.matrix <- makeContrasts(AVD-Control,levels=design)

library(futile.logger)

fit2 <- contrasts.fit(fit, contrast.matrix)

fit2 <- eBayes(fit2)

alldiff <- topTable(fit2,adjust.method="fdr",coef=1,number=50000)

alldiff_new <- na.omit(alldiff)

write.csv(alldiff_new,file="alldiff_DNmrna_expr_3sets_batchnew.csv")

**Figure 3**

**WGCNA working sheets:**

rm(list = ls())

library(WGCNA)

options(stringsAsFactors = FALSE)

enableWGCNAThreads()

setwd("")

samples=read.csv('target_3ets_wgcna.txt',sep = '\t',row.names = 1)

expro=read.csv('mrna_expr_3sets_batch.txt',sep = '\t',row.names = 1)

dataExpr=expro

m.mad <- apply(dataExpr,1,mad)

dataExprVar <- dataExpr[which(m.mad >

max(quantile(m.mad, probs=seq(0, 1, 0.25))[2],0.01)),]

dataExpr <- as.data.frame(t(dataExprVar))

gsg = goodSamplesGenes(dataExpr, verbose = 3)

if (!gsg$allOK){

# Optionally, print the gene and sample names that were removed:

if (sum(!gsg$goodGenes)>0)

printFlush(paste("Removing genes:",

paste(names(dataExpr)[!gsg$goodGenes], collapse = ",")));

if (sum(!gsg$goodSamples)>0)

printFlush(paste("Removing samples:",

paste(rownames(dataExpr)[!gsg$goodSamples], collapse = ",")));

dataExpr = dataExpr[gsg$goodSamples, gsg$goodGenes]

}

nGenes = ncol(dataExpr)

nSamples = nrow(dataExpr)

dim(dataExpr)

powers = c(c(2:10, by=1), seq(from = 12, to=20, by=2))

sft = pickSoftThreshold(dataExpr, powerVector = powers, verbose = 5)

par(mfrow = c(1,2));

cex1 = 0.9;

plot(sft$fitIndices[,1], -sign(sft$fitIndices[,3])*sft$fitIndices[,2],

xlab="Soft Threshold (power)",ylab="Scale Free Topology Model Fit,signed R^2",type="n",

main = paste("Scale independence"));

text(sft$fitIndices[,1], -sign(sft$fitIndices[,3])*sft$fitIndices[,2],

labels=powers,cex=cex1,col="red");

abline(h=0.85,col="red")

plot(sft$fitIndices[,1], sft$fitIndices[,5],

xlab="Soft Threshold (power)",ylab="Mean Connectivity", type="n",

main = paste("Mean connectivity"))

text(sft$fitIndices[,1], sft$fitIndices[,5], labels=powers, cex=cex1,col="red")

power = sft$powerEstimate

sft$powerEstimate

net = blockwiseModules(dataExpr, power = 5, maxBlockSize = 6000,

TOMType = "unsigned", minModuleSize = 50,

reassignThreshold = 0, mergeCutHeight = 0.25,

numericLabels = TRUE, pamRespectsDendro = FALSE,

saveTOMs = TRUE,

saveTOMFileBase = "AS-green-FPKM-TOM",

verbose = 3)

table(net$colors)

moduleLabels = net$colors

moduleColors = labels2colors(net$colors)

table(moduleColors)

MEs = net$MEs;

geneTree = net$dendrograms[[1]];

save(MEs, moduleLabels, moduleColors, geneTree,

file = "AS-green-FPKM-02-networkConstruction-auto.RData")

moduleLabelsAutomatic = net$colors

moduleColorsAutomatic = labels2colors(moduleLabelsAutomatic)

moduleColorsWW = moduleColorsAutomatic

MEs0 = moduleEigengenes(dataExpr, moduleColorsWW)$eigengenes

MEsWW = orderMEs(MEs0)

modTraitCor = cor(MEsWW, samples, use = "p")

colnames(MEsWW)

modlues=MEsWW

modTraitP = corPvalueStudent(modTraitCor, nSamples)

textMatrix = paste(signif(modTraitCor, 2), "\n(", signif(modTraitP, 1), ")", sep = "")

dim(textMatrix) = dim(modTraitCor)

labeledHeatmap(Matrix = modTraitCor, xLabels = colnames(samples), yLabels = names(MEsWW), cex.lab = 0.5, yColorWidth=0.01,

xColorWidth = 0.03,

ySymbols = colnames(modlues), colorLabels = FALSE, colors = blueWhiteRed(50),

textMatrix = textMatrix, setStdMargins = FALSE, cex.text = 0.5, zlim = c(-1,1)

, main = paste("Module-trait relationships"))

**Figure 7**

**LASSO working sheets:**

setwd("")

library("glmnet")

expr <- read.table("luster_DEGs.txt", header = T,row.names=1,sep="\t")

x <-2^expr

x <- t(x)

x <- as.matrix(x)

target <- read.table("target_3ets_wgcna.txt", header = T,row.names=1,sep="\t")

y <- as.numeric(target[,2])

set.seed(666)

train <- sample(1:nrow(x), nrow(x)*2/3)

test <- (-train)

r2 <- glmnet(x = x[train,], y = y[train], family = "binomial", alpha = 1)

print(r2)

plot(r2, xvar = "lambda",label = TRUE)

r2.cv <- cv.glmnet(x = x, y = y, family = "binomial", alpha = 1, nfold = 10, type.measure="auc")

plot(r2.cv)

r2.cv$lambda.min

r2.cv$lambda.1se

r2.1se <- glmnet(x = x, y = y, family = "binomial", alpha = 1, lambda = r2.cv$lambda.1se)

r2.min <- glmnet(x = x, y = y, family = "binomial", alpha = 1, lambda = r2.cv$lambda.min)

print(r2.1se)

print(r2.min)

predict(r2.cv, s=r2.cv$lambda.1se, type="coefficients")

predict(r2.cv, s=r2.cv$lambda.min, type="coefficients")

coefficients<-coef(r2.cv,s=r2.cv$lambda.1se)

Coefficients

**Random Forest working sheets:**

library(randomForest)

mydata<-read.table("",header=TRUE,sep=",",row.names = 1)

mydata$AVD<-factor(mydata$AVD)

mydata.rf<-randomForest(AVD~.,data=mydata,importance=TRUE,proximity=TRUE)

importance(mydata.rf,type=2)

varImpPlot(mydata.rf, main = "variable importance")

pre_ran <- predict(mydata.rf,newdata=mydata)

**Figure 8**

**Nomogram working sheets:**

d1<-read.table("....txt",header=T,sep=",",row.names = 1)

library(car)

f1<-glm(AVD ~ MMP9 + SLPI,data=d1,family=binomial())

d1$score<-predict(f1, newdata = d1)

library(rms)

ddist<-datadist(d1)

options(datadist="ddist")

f1<-lrm(AVD ~ ,data=d1)

nomo<-nomogram(f1,fun = plogis,fun.at = c(seq(.1,.9, by=.1),0.95,0.99,0.01,0.05),lp=F,funlabel = "Probability of CAVD")

plot(nomo,xfrac=.4)

library(pROC)

roc1<-roc(d1$AVD,d1$score)

auc(roc1)

ci.auc(roc1)

plot(roc1, col="red")

ft<-lrm(AVD ~ MMP9 + SLPI,data = d1,x=T,y=T)

cal1<-calibrate(ft,group=d1$AVD)

opar<-par(no.readonly=T)

par(pin=c(3,3))

plot(cal1,xlim = c(0,1.0),ylim = c(0,1.0),xlab = "Nomogram predicted probability",ylab = "Actual CAVD rate",subtitles=F,legend=F)

par(opar)

setwd("...")

source("dca.R")

f1<-glm(AVD ~ MMP9 + SLPI, data = d1, family=binomial(link="logit"))

d1$Nomogram= predict(f1, type="response")

dca(data=d1, outcome="CAVD", predictors="Nomogram",xstart=0.01, xstop=0.99, xby=0.02, ymin=-0.05, probability=T,smooth = T)

**Figure 9**

**Cibersort working sheets:**

library(CIBERSORT)

data(LM22)

setwd("...")

mixed_expr=read.csv("FAmerge_DN_matrix.txt",header=TRUE,sep="\t",row.names = 1)

mixed_expr=2^mixed_expr

results <- cibersort(sig_matrix = LM22, mixture_file = mixed_expr,perm = 1000, QN = F)

write.table(results,"results_immune infiltration.txt",sep="\t")

**Correlation analysis:**

setwd("...")

expr_data=read.csv("....txt",header=TRUE,sep="\t",row.names = 1)

immu_data=read.csv("results_immune infiltration.txt",header=TRUE,sep="\t",row.names = 1)

sig_gene <- c("...")

library(psych)

x <- expr_data[,sig_gene]

y <- immu_data

library(psych)

d <- corr.test(x,y,use="complete",method = 'spearman')

r <- d$r

p <- d$p

library(ggcorrplot)

ggcorrplot(t(d$r), show.legend = T,

p.mat = t(d$p.adj), digits = 2, sig.level = 0.05,insig = 'blank',lab = T)
